# Student loan debt and financial education: a qualitative analysis of resident perceptions and implications for resident well-being

**DOI:** 10.1080/10872981.2022.2075303

**Published:** 2022-05-18

**Authors:** Cameryn C. Garrett, Ronda L. Doonan, Casey Pyle, Michelle B. Azimov

**Affiliations:** a Graduate Medical Education, Community Memorial Health System, Ventura, California, USA; b Orthopaedic Surgery Residency Program, Community Memorial Health System, Ventura, California, USA

**Keywords:** well-being, debt, student loans, medical education, qualitative methods

## Abstract

High educational debt is prevalent among resident physicians and correlates with adverse well-being outcomes, including symptoms of stress and burnout. Residents also report low financial literacy levels, affecting financial well-being. Understanding resident viewpoints toward financial well-being initiatives is crucial to develop targeted resident financial well-being programs. This study aims to examine residents’ experiences financing their medical education and how these experiences influence well-being and attitudes toward financial education in residency. We recruited residents from a Southern California health system with residency programs in Family Medicine, Internal Medicine, General Surgery, Orthopaedic Surgery, and Psychiatry. We contacted residents by email and text message to participate in semi-structured interviews. We conducted interviews from October 2020 to March 2021 and analyzed 59 resident interviews using reflexive thematic analysis. Among residents, 76% (45/59) had ≥ $200,000 in student loans. Residents perceived mounting medical education debt as unfairly burdensome for trainees engaged in socially beneficial work, leaving residents feeling undervalued – a feeling heightened by the stressors of the COVID-19 pandemic – and hampering well-being. Compartmentalizing debt attenuated financial stressors but often made financial education seem less pressing. A subset of residents described how financial planning restored some agency and enhanced well-being, noting that protected didactic time for financial education was crucial. Resident interviews provide practical guidance regarding designing financial education sessions. Desired education included managing debt, retirement planning, and the business of medicine. How residents framed educational debt and their degree of financial literacy impacted their well-being and sense of agency. Residents proposed that residency programs can aid in stress mitigation by providing residents with skills to help manage debt and plan for retirement. To reduce clinician indebtedness, this approach needs to occur in tandem with systemic changes to financing medical education.

## Introduction

High educational debt is prevalent among resident physicians [1,2] and correlates with adverse well-being outcomes, including symptoms of burnout and stress [3–5], decreased quality of life [4], and reduced satisfaction with work-life balance [4]. These symptoms affect personal and professional well-being and can negatively affect the quality of patient care [6]. As outlined by the Accreditation Council for Graduate Medical Education (ACGME), graduate medical education programs have a responsibility to promote resident well-being [7]. As such, program leadership has a vested interest in exploring and addressing factors influencing physician well-being.

While medical associations have highlighted financial health as important to physician well-being [8], residents commonly display low levels of financial literacy [9,10]. Residents are rarely provided with formal financial training yet report interest in education on topics including debt management and retirement plannin [10–12] Improving one’s financial literacy through education in the training and work environment can positively influence financial planning behaviors and decisions [13].

Despite evidence of a link between financial education and physician well-being, knowledge of how resident attitudes toward educational debt influence financial planning behaviors remains limited. Residents report varied attitudes and behavioral approaches to financial management [14], with limited qualitative research suggesting high debt levels can increase resident feelings of helplessness and deemphasize budgeting behaviors as sums owed can feel insurmountable [12]. Given the importance of physician well-being in graduate medical education, more qualitative research is needed to further examine the emotional and psychological impact of educational debt on residents’ experiences and views towards financial well-being initiatives.

Our multispecialty study aims to learn more about residents’ experiences financing their education through student loans and how these experiences affect their well-being and attitudes toward financial education in residency.

## Materials and methods

We report the study methods in accordance with the Consolidated Criteria for Reporting Qualitative Research (COREQ) [15].

### Setting and participants

We enrolled participants for our qualitative study from a convenience sample of residents at Community Memorial Health System (CMHS). CMHS is an urban Southern California community health system with five residency programs: Family Medicine (FM), Internal Medicine (IM), General Surgery (GS), Orthopaedic Surgery (OS), and Psychiatry (Psych). We took a pragmatic approach to recruitment, considering time and other project constraints and data content. To gain diverse perspectives, we sought residents in multiple specialties, given evidence of an association between educational debt and specialty choice [3], as well as by gender and year of training.

### Data collection and analysis

We developed an interview guide based on an iterative literature review and piloted interviews with three medical students for clarity and flow. Interviews were selected over focus groups given the potentially sensitive nature of personal finances and mental wellbeing. We recruited residents by email and text message. Residents interested in participating contacted C.G. and were sent a Plain Language Statement. Before the formal interview began, C.G. explained the reasons for conducting the study, and each participant provided informed verbal consent before commencing the interview. C.G., an experienced qualitative researcher with a Ph.D. in Public Health, conducted all the interviews. C.G. is a Graduate Medical Education staff member but did not have a supervisory role over any potential participants. C.G. conducted interviews by telephone or video call from October 2020 to March 2021. Participants were interviewed one-on-one at home or their workplace, depending on their preference and availability. In the interview, we asked participants about how they financed their education, their perspectives on whether the ways they financed their education influenced personal and professional decisions, and their views on financial education during residency. For our current analysis, we excluded from the dataset 12 residents who reported zero student loan debt as our focus was the experiences of residents with student loan debt. The average interview length was 17 minutes. One interview was cut short because the participant was on call and had to attend to clinical duties. Due to that resident’s time constraints, we were unable to schedule a follow-up call to gain that resident’s views on desired financial education. One additional resident declined to participate after an interview had been scheduled due to patient care responsibilities. Participants were offered a $5 coffee voucher as compensation. All interviews were audio-recorded and transcribed verbatim, removing minor repetition and pauses for readability [16]. One resident declined permission to record so C.G. took detailed notes. C.G. de-identified the transcripts and gave each participant a code number.

We conducted our data collection and analysis concurrently, with the richness of the dataset in relation to our study aims determining when no further interviews were required [16]. We employed thematic analysis to identify shared patterns of meaning across residents’ accounts. We adopted an experiential orientation to our analysis, staying close in the analysis to meanings and understandings expressed by residents and conceptualized language as intentional [16]. We analyzed interviews using a six-phased reflexive thematic analysis [16,17]. Our analysis was guided by a critical realist perspective [16,18], which acknowledges that our representations of reality are mediated by social and psychological factors. We familiarized ourselves with the dataset through listening to audio files and reading and re-reading the transcripts. Coding involved multiple readings of the dataset; we generated codes with a more inductive (bottom up) orientation to data through multiple readings of the dataset, as opposed to a more deductive (top down) theory-driven orientation. We acknowledge, as outlined by Braun and Clarke (2022), that our inductive orientation to data ‘is never “pure” because of what we bring to the data analytic process, as theoretically embedded and socially positioned researchers’ (pg. 56). We constructed initial themes based on shared patterns of meaning and utilized visual mapping to examine how initial themes related to one another. We developed and reviewed candidate themes, examining the analysis in relation to the coded extracts and then the full dataset. We then refined, defined, and named themes, with repeated reference to the dataset. The sixth phase was writing the report. We utilized QSR NVivo software for data management and to keep an electronic trail of the analysis, including interviewer notes after interviews, a record of the codes, thematic maps, definitions of key themes, and reflective entries regarding the analysis process. C.G. led the analysis and R.D. and M.A. reviewed a subset of transcripts; the reflective and collaborative analysis by multiple authors enabled richer data interpretation [19].

### Team reflexivity

Our research team consisted of multidisciplinary clinicians and an experienced qualitative researcher. The physicians financed their medical education through student loans, and one author recently completed his residency at CMHS (C.P.). R.D. oversees the resident well-being curriculum, and C.P. provides informal financial well-being mentoring to residents. Each author held the view that financial education could be a useful component of residency wellbeing initiatives. Throughout the process, we reflected on how our multidisciplinary training, educational debt experiences, and engagement in resident well-being activities informed our research and influenced how we interpreted the data.

Community Memorial Health System Institutional Review Board approved the study (#2019-HSR002E).

## Results and thematic interpretation

Participants comprised fifty-nine residents who completed semi-structured interviews; 76% (45/59) had ≥ $200,000 in educational debt (Table 1). We identified three key themes: 1) daunting debt for socially beneficial work is unfairly burdensome, 2) compartmentalizing debt makes financial education seem less pressing, 3) financial literacy is one way to assert control over one’s financial future. We conclude by providing practical guidance for program leadership regarding designing financial education sessions.

**Table 1.** Characteristics of the 59 participants

Characteristic	n (%)
**Specialty**	
Family Medicine	14 (24)
Internal Medicine	15 (25)
General Surgery	10 (17)
Orthopaedic Surgery	17 (29)
Psychiatry	3 (5)
**Gender**	
Female	17 (29)
Male	42 (71)
**Age**	
25 to 29 years	18 (31)
30 to 34 years	35 (59)
35 to 39 years	4 (7)
40+ years	2 (3)
**Level of training**	
PGY-1	21 (36)
PGY-2	10 (17)
PGY-3	15 (25)
PGY-4	7 (12)
PGY-5	6 (10)
**Medical school**	
Osteopathic	55 (93)
Allopathic	4 (7)
**Educational debt**	
≥ $100,000	52 (88)
≥ $200,000	45 (76)
≥ $300,000	37 (63)
Abbreviations: PGY, postgraduate year	

### Daunting debt for socially beneficial work is unfairly burdensome

Residents frequently conceptualized student loans within the American medical education system as a *‘necessary evil’* (P48, OS), an unwelcome yet obligatory step to enable one’s professional goals. Their understanding of indebtedness was often anchored in the self-sacrifice they perceived society expects of physicians. Residents described sacrificing their early adulthood to long hours of training, often absent from activities with family and friends. The forced choice of accruing high debt to pursue a profession dedicated to helping others shaped their identity and compounded feelings of self-sacrifice.

*“I owe more than some people on houses just from my education. You get into this field to help people if you can, and I feel it comes at a very big cost. It’s this whole mindset and system that you have to sacrifice something, and you have to sacrifice so much of yourself to help people … It’s just the overarching system that makes us believe that we have to sacrifice our time, our well-being, our emotional well-being, and then financially too, that we have to take on this debt. Medical education is not a priority to subsidize or make easy for a lot of people to obtain.” (P58, GS)*

Residents generally portrayed high levels of debt as a reflection of society’s underinvestment in a public good. Steep educational costs to pursue clinical training, and in turn high debt levels for young physicians, were an inequity they felt existed given the time, commitment, and sacrifices trainees undertake to help care for society. High debt levels left residents feeling undervalued and portended negative mental health outcomes.

*“It’s really atrocious how much we can get charged … Maybe I was naïve to not think of the cost more before. But I think that part of it [how debt influences well-being] is highlighted by the fact there’s so much burnout with the job, or job dissatisfaction … [If I didn’t have student loan debt,] it would make it so much better because it would make it feel like what I do every day is fair.” (P14, IM)*

*“It’s [the debt is] not because we’re irresponsible or spending more than we could afford. It’s because we wanted to become educated and have this career … It [debt] will contribute to burnout. I think a lot of doctors will try to take on more jobs and will burn out faster, and I think not having financial security and being overworked will lead to possible self-harm ideas.” (P27, FM)*

Medical debt compounded the stress and time demands of the profession and could unfavorably tilt physicians’ work life balance. Residents framed loan repayments as restricting future career and family options as they prioritized meeting debt obligations; as one resident shared, *‘it really limits you, what, where you wanna work and pulls and tugs at why you love medicine and why you want to practice’* (P32, IM). Notably, however, a small proportion of residents described student loans in more positive terms; employing a neoliberal framing of education, they conceptualized student loans as a personal capital investment [20]. Orthopaedic surgeons and some general surgeons were more likely than primary care residents to position loans as a rational financial investment when considering their earning potential and employment opportunities. Noting their projected salaries, these surgical residents described their careers as having *‘a good enough return on investment’* (P38, OS) to minimize the debt burden.

The COVID-19 pandemic further shaped participants’ views on debt and at times compounded frustration as it affected residents’ lives inside and outside the hospital, with stressful work hours, decreased family time, and decreased ability to decompress with friends or travel. The pandemic also placed a spotlight on the societal importance of the medical field, and in this context, residents’ high levels of debt often did not sit neatly with their daily sacrifices as frontline workers. While Internal Medicine residents cared for a significant portion of the influx of hospitalized patients, the COVID burden was reported across specialties at the institution, particularly as the interviews were conducted before a COVID-19 vaccine was widely available, and residents were concerned not only for their own well-being but for that of their family members:

*“Especially with COVID, I mean we’re putting our own lives on the line and putting my family members at risk and to have this much debt is stupid. It’s just totally stupid. It’s upsetting … I do feel like we deserve some sort of forgiveness and not in the form of a $1,200.00 check or forbearance.” (P26, GS)*

The amplified risks to their health and safety while caring for others in a pandemic provided context to validate further the perception that medical education was a societal good worth additional government investment. The pandemic exacerbated many residents’ sense of being undervalued; residents expressed a desire to be recognized for honoring their societal duty as frontline workers by what they perceived as society’s reciprocal obligation to invest in their medical education beyond temporary financial measures such as the Congressional Coronavirus Aid, Relief, and Economic Security (CARES) Act.

Participants also regularly compared high interest rates for their medical education loans with comparatively low rates for home loans.

*“It’s crazy that we have an interest rate that’s so high, especially if we’re going through a government route … We’re doing something that’s great for the community, something that is needed, but it’s almost like we get punished.” (P24, FM)*

Additional debt accruing while still in training led some residents to feel like they were assuming a disproportionate burden to protect others’ health and well-being. High tuition costs coupled with high interest rates raised the specter of an unfair system where others were profiting at their expense.

### Compartmentalizing debt makes financial education seem less pressing

A common strategy to attenuate financial stress from obligatory student loans was to compartmentalize debt. Residents explained that while debt would considerably affect future decisions, such as timing of homeownership and job considerations, many pushed debt to the back of their minds as they focused on completing residency.

*“[I] kind of try not to think about it [debt] as a coping mechanism and then also because we’re so busy with everything else that it’s a burden that we don’t want to deal with. It’s easy to put in the back, or the rearview mirror, not think about it when it’s not really affecting me right now.” (P01, FM)*

*“It [debt] is a big, lingering, black cloud, you know, that, at the end of the day, I know will have to be paid off … I think it’s just a matter of compartmentalizing … and saying, okay, we’ll deal with it when time comes.” (P12, OS)*

Structural factors such as lower student loan repayments during residency facilitated residents in compartmentalizing debt. The governmental pause on federal loan repayments and interest in response to the COVID-19 pandemic [21] further minimized the focus on debt repayment.

While compartmentalizing debt offered residents a coping strategy, it often made financial education during training seem less relevant as debt repayment was categorized largely as a future worry. High sums owed for medical education could make debt seem like *‘Monopoly money’* (P56, FM), a fanciful number not requiring immediate attention. For most, financial education was deprioritized, cast as an activity that would *‘be great to do if I had more time’* (P21, IM). While residents expressed interest in improving their financial knowledge, participants across different years of training, debt levels, and in both medical and surgical specialties, described focusing their attention during training on what they saw as more immediate professional and personal needs.

*“Whenever you’re a college student, the only thing you focus on is getting into medical school. Whenever you’re a medical student, the only thing you focus on is getting into residency. And now that I’m a resident, the only thing I’m focusing on is getting through residency and trying to graduate all my tests, and to learn everything, learn all the procedures, all the surgical skills, to become an attending. And there’s really no time at all in that to study anything financially.” (P50, OS)*

As financial education was rarely framed as an immediate need, the inference was that any optional course offered during free time would fall to the wayside. As such, residents recommended providing financial education during structured didactics to increase uptake.

### Financial literacy is one way to assert control over one’s financial future

While not a panacea, for a subset of residents particularly in Orthopaedic Surgery and Family Medicine, enhancing financial literacy helped mitigate stress connected to student loans by enabling them to assert some control over their financial future. As one resident illustrated, devising a specific financial plan can reduce stress by providing strategies to actively manage debt:

*“When I was younger, in med school maybe, and I had a weaker, more tenuous grasp on personal finance maybe, it [debt] was kind of a weight on my conscience. But I feel like I have a good enough grasp on where I am now that it doesn’t bother me very much … [What changed] was [a physician financial education] course. That was a huge turning point for my ability to kind of get my arms around what I have to do and how to do it.” (P19, OS)*

Increasing financial knowledge helped to restore agency and made debt-related stress more tolerable.

The success of financial literacy as a debt management strategy was shaped by access to mentors and future earning potential. Enthusiastic adopters of financial education acted as mentors and nudged peers, particularly within the Orthopaedic Surgery residency program, to actively manage their finances.

*“It [my financial knowledge] definitely increased recently, which is good. It’s mostly been through fellow residents … bein’ like, ‘Hey, have you set this [employee retirement account] up? Do you have your Roth set up? Are you making contributions? Is [the hospital] matching?’” (P42, OS)*

Financially savvy attendings also provided guidance:

*“I really knew nothing about financial education, and he [the attending] talked to me about opening up a Roth IRA and talked to me about 529 [education savings plan] … He gave me, I guess, a foundation to start with, and I kinda just took off and built on that foundation.” (P23, OS)*

Orthopedic Surgery residents also acknowledged in the interviews the benefit of their specialty’s high earning potential to help repay educational debts. Residents in this specialty both extolled the mental health benefits of developing a financial plan and noted that with careful planning and judicious spending, one could be debt free shortly after residency.

In medical specialties, residents’ debt-to-earning potential ratios often meant many more years of working before paying loans off. Even after creating a financial plan, debt could still loom large over one’s mental landscape; in lower-paying specialties, residents described at times feeling as if they might take *‘this [medical education] payment to the grave’* (P02, FM). As one Family Medicine resident explained, while creating a financial plan instilled agency to assert some control over one’s finances, the strategy had limits and could only attenuate the mental health impact of high educational debt.

*“[Debt is] almost like a monkey on your back, that you always have that pressure of something there … It’s kind of like a consistent anxiety with knowing that you have so much money to pay back … It [creating the financial plan] makes me feel like I have better control over things, at least financially, so that kind of lowers the overall stress level because I feel like you know I’m doing something about it [the debt], or I know what I’m going to do about it.” (P17, FM)*

Enhancing one’s financial literacy and prudent spending and saving cannot alone erase hundreds of thousands of dollars of loans. Residents suggested financial education and budgeting not as an ultimate remedy but as one tool to lower a stressor of residency and make paying off one’s debt not seem *‘so impossible’* (P32, IM). Some residents expressed frustration that residency educational goals and objectives did not include financial education, given the link they saw between debt, mental health, and burnout and the perceived usefulness of financial education in addressing these issues.

*“I just think it [financial education] should be a mandatory part of training. And I really don’t understand why, like, you know, ACGME hasn’t implemented this. Like, it’s well known how much, you know, distress this causes to residents being under so much pressure, like financial pressure.” (P23, OS)*

Residents framed financial education as a tool to mitigate financial stress and advocated for residency programs to provide them with skills to help manage debt and plan for their financial future, much like ACGME provides achievable Milestones to guide the progressive acquisition of skills for core competencies.

### Practical guidance for resident financial education

**Figure 1.**
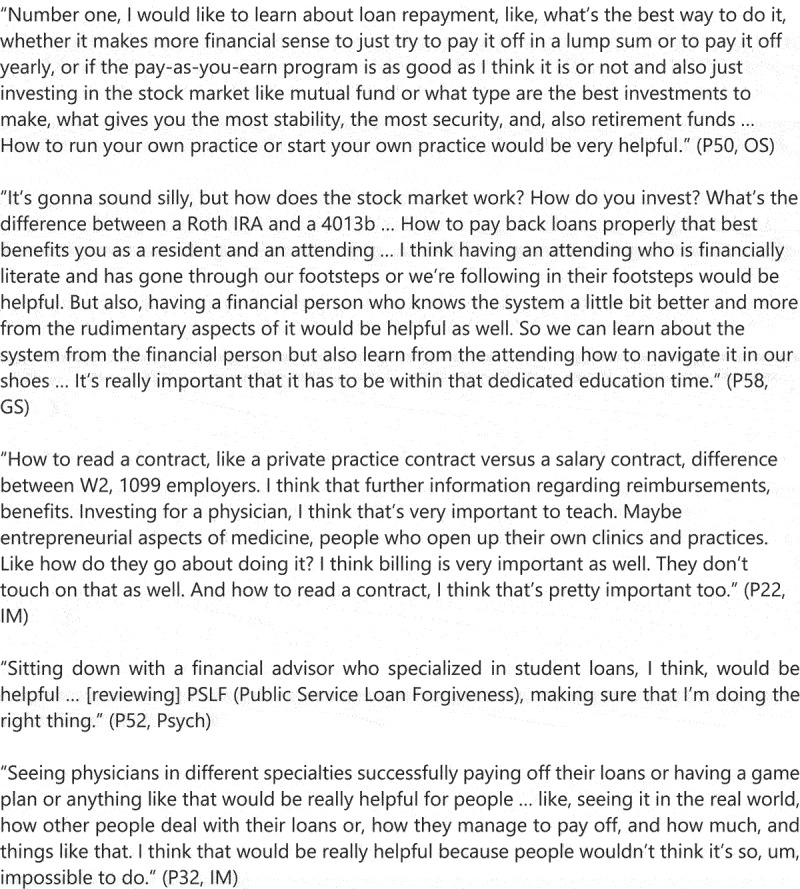
Illustrative quotations of desired financial education.

The resident interviews offer some practical guidance regarding designing financial education sessions (see Figure 1 for illustrative quotations of desired financial education):

- Provide interactive sessions during protected didactic time led by: a) attending physicians who had ‘walked in their shoes’ and successfully managed student loan debt and b) financial advisors knowledgeable about physician student loan debt and projected future incomes.
- Focus on topics such as student loan repayment strategies and Public Service Loan Forgiveness; budgeting and resource allocation; retirement planning (retirement accounts and investing); and the business of medicine (billing and coding, contract negotiation, and running a private practice).
- Offer individualized sessions with a financial advisor.
- Record group sessions so residents on night or away rotations can access content.

At our institution, we recently piloted several new financial education opportunities, including life after residency financial education mentoring discussions with financial savvy attendings, financial education roundtables and individual consultations with financial advisors, engagement with institutional resources such as the Association of American Medical College’s FIRST (Financial Information, Resources, Services and Tools) guidance, and provision of physician financial education reference materials. Our financial education curriculum will be further tailored based on resident feedback.

## Discussion

Residents across specialties perceived mounting medical education debt as unfairly burdensome for trainees engaged in socially beneficial work, leaving residents feeling undervalued – a feeling heightened by the stressors of the COVID-19 pandemic – and hampering well-being. A subset of residents described how financial planning restored some agency and enhanced their financial well-being. Residents provided recommendations on how programs can provide education to foster financial education, including guidance on types of speakers and preferred content.

The medical education community is increasingly focused on clinician burnout and physician well-being [6]. These concerns have been further heightened in the COVID-19 pandemic, which placed additional stressors on physicians and re-emphasized the idea that physicians are self-sacrificing and heroic [22]. Cox [23] posits that the heroism narrative overlooks the role of reciprocity in physicians’ duty to treat, which can negatively impact physicians’ mental health. As Cox explains, ‘the hero narrative fails to remind the public and healthcare institutions of their own moral duties, as in its focus on individual healthcare workers’ selfless sacrifice it does not recognise that their duty to treat is irrevocably tied to reciprocal societal obligations.’ [23] In our interviews, residents suggested that society had a reciprocal duty towards physicians to minimize medical education debt burden. Earlier research reported that high medical education debt resulted in medical students feeling cynical and less altruistic [24], eroding that sense of altruism at the onset of professional training. Despite an initial sense of reciprocity, residents in our study expressed how, particularly during a pandemic, high debts – typically above $300,000 – to pursue medical education compounded their feelings of self-sacrifice and negatively affected their well-being. High interest rates for government educational loans while in training were also viewed as betraying the reciprocal duty that society has towards physicians. While residents appreciated the pause on loan repayments and interest via the CARES Act [21], it provides only temporary relief.

While high levels of clinician indebtedness should be addressed at a policy level, one organizational-level strategy to promote physician well-being is incorporating debt management and financial well-being into the formal curriculum [8,25]. Residents report high levels of interest in financial education [10,26]; indeed some residents in our study were actively engaged in improving their financial literacy. These residents described how financial planning enhanced a sense of control over their financial futures and engendered well-being. Notably, in the Orthopaedic Surgery program, educated peer and attending mentors fostered an environment of intellectual curiosity around financial well-being and facilitated engagement in financial education. Senior residents and attending physicians provided ‘curbside’ financial education and directed residents towards trusted physician financial resources. These mentors helped to transform what could be considered a ‘black box’ into a more manageable, approachable topic. Additional research could examine the role of mentorship as one element of a financial education curriculum during residency training.

Programs should recognize that financial education may not always be considered a priority among residents. Young et al [12]. reported that Emergency Medicine residents often deprioritized financial education, perceiving it as a topic they could delay until after graduation. Our study extended this finding to surgical and medical residents, with participants reporting that financial education during residency often seemed less pressing compared with more immediate personal and professional responsibilities. These findings provide evidence that financial education courses may be more successful if provided during mandatory structured didactics instead of as optional courses in which some residents may be unlikely to participate. Our study adds to the literature by providing practical guidance to program leadership looking to augment resident financial well-being.

In our study, we used a convenience sample of residents from one health system, and as such, one should be wary of interpreting our findings as a generalized account of residents’ experiences. While our study included diversity in specialty, gender, and year of training, our sample was limited to a single institution, and most residents in our programs trained in osteopathic medical schools. Median medical education debt is higher among osteopathic graduates compared with allopathic graduates ($275,000 vs. $200,000) [1,2]; increased levels of debt may further impact well-being [27]. Future studies could include more geographic diversity and examine resident experiences across institutions; some of the findings regarding financial stress may have been attenuated by only sampling residents in an institution in California, a state with high housing prices and state employment laws that restrict hospital physicians’ eligibility for Public Student Loan Forgiveness.

More mixed methods research is needed to investigate the type and structure of financial education interventions to successfully impact residents’ financial knowledge and well-being.

## Conclusions

Financial literacy and how residents framed educational debt affected both residents’ well-being and sense of agency. Residents proposed that residency programs can aid in financial stress mitigation by providing them with skills during formal protected didactic time to help manage debt and plan for retirement.

## Data Availability

The data that support the findings of this study are available from the corresponding author, C.G., upon reasonable request, and with approval from the Community Memorial Health System Institutional Review Board.

## References

[1] American Association of Colleges of Osteopathic Medicine. 2019-2020 Academic Year Graduating Seniors Survey Summary Report. https://www.aacom.org/docs/default-source/data-and-trends/aacom-2019-2020-graduating-seniors-survey-summary-report.pdf?sfvrsn=406a0897_4. Accessed 2021 Dec 14.

[2] Association of American Medical Colleges. *Medical School Graduation Questionnaire: 2020 All Schools Summary Report*. https://www.aamc.org/media/46851/download. Cited 2021 Dec 14.

[3] Pisaniello MS, Asahina AT, Bacchi S, et al. Effect of medical student debt on mental health, academic performance and specialty choice: a systematic review. BMJ Open. 2019;9(7):e029980. 10.1136/bmjopen-2019-029980 PMC6609129 31270123

[4] West CP, Shanafelt TD, Kolars JC. Quality of life, burnout, educational debt, and medical knowledge among internal medicine residents. JAMA. 2011;306(9):952–8. 21900135 10.1001/jama.2011.1247

[5] Zhou AY, Panagioti M, Esmail A, et al. Factors associated with burnout and stress in trainee physicians: a systematic review and meta-analysis. JAMA Network Open. 2020;3(8):e2013761. 32809031 10.1001/jamanetworkopen.2020.13761 PMC7435345

[6] National Academies of Sciences, Engineering, and Medicine. 2019. Taking action against clinician burnout: A systems approach to professional well-being. Washington, DC: The National Academies Press. 10.17226/25521 31940160

[7] The Accreditation Council for Graduate Medical Education. ACGME Common Program R equirements. https://www.acgme.org/what-we-do/accreditation/common-program-requirements. Cited 2021 Dec 10.

[8] Okanlawon T. *Resident and Fellow Burnout: Create a Holistic, Supportive Culture of Well-Being*. American Medical Association. https://edhub.ama-assn.org/steps-forward/module/2702511. Cited 2021 Nov 18.

[9] Ahmad FA, White AJ, Hiller KM, et al. An assessment of residents’ and fellows’ personal finance literacy: an unmet medical education need. Int J Med Educ. 2017;8:192–204. 28557777 10.5116/ijme.5918.ad11 PMC5457786

[10] McKillip R, Ernst M, Ahn J, et al. Toward a resident personal finance curriculum: quantifying resident financial circumstances, needs, and interests. Cureus. 2018;10(4):e2540. 29951347 10.7759/cureus.2540 PMC6019332

[11] Shappell E, Ahn J and Ahmed N, et al.$3$2 (2018). Personal Finance Education for Residents: a Qualitative Study of Resident Perspectives. AEM Educ Train, 2(3), 195–203. 10.1002/aet2.10090 30051089 PMC6050061

[12] Young TP, Brown MM and Reibling ET, et al.$3$2 (2016). Effect of Educational Debt on Emergency Medicine Residents: a Qualitative Study Using Individual Interviews. Ann Emerg Med, 68(4), 409–418. 10.1016/j.annemergmed.2016.04.013 27181080

[13] Lusardi A. Financial literacy and the need for financial education: evidence and implications. Swiss J Econ Stat. 2019;155(1):1.

[14] Wong R, Ng P, Bonino J, et al. Financial attitudes and behaviors of internal medicine and internal medicine-pediatrics residents. J Grad Med Educ. 2018;10(6):639–645. 30619520 10.4300/JGME-D-18-00015.1 PMC6314372

[15] Tong A, Sainsbury P, Craig J. Consolidated criteria for reporting qualitative research (COREQ): a 32-item checklist for interviews and focus groups. Int J Qual Health Care. 2007;19(6):349–357. 17872937 10.1093/intqhc/mzm042

[16] Braun V, Clarke V. Thematic analysis: a practical guide. Thousand Oaks CA: SAGE Publications Inc; 2022.

[17] Braun V, Clarke V. Using thematic analysis in psychology. Qual Res Psychol. 2006;3(2):77–101.

[18] Willig C. Introducing qualitative research in psychology. 3rd ed. Maidenhead England: Open University Press; 2013.

[19] Braun V, Clarke V. Reflecting on reflexive thematic analysis. Qual Res Sport Exerc Health. 2019;11(4):589–597.

[20] Brown W. Neoliberalized knowledge. *Hist Present*. 2011;1(1):113–129.

[21] Federal Student Aid. COVID-19 Emergency Relief and Federal Student Aid. Office of the Department of Education. https://studentaid.gov/announcements-events/covid-19. Cited 2021 Nov 23.

[22] Hughes MT, Rushton CH. Ethics and well-being: the health professions and the COVID-19 pandemic. Acad Med. 2022 97 3 S98–S103 . published online ahead of print, 16 Nov https://journals.lww.com/academicmedicine/Fulltext/2022/03001/Ethics_and_Well_Being__The_Health_Professions_and.16.aspx. DOI:10.1097/acm.0000000000004524. 34789657 PMC8855760

[23] Cox CL. ‘Healthcare heroes’: problems with media focus on heroism from healthcare workers during the COVID-19 pandemic. J Med Ethics. 2020;46(8):510–513. 32546658 10.1136/medethics-2020-106398 PMC7316119

[24] Phillips JP, Wilbanks DM, Salinas DF, et al. Educational debt in the context of career planning: a qualitative exploration of medical student perceptions. Teach Learn Med. 2016;28(3):243–251. 27152504 10.1080/10401334.2016.1178116

[25] Dyrbye L, Shanafelt T. A narrative review on burnout experienced by medical students and residents. Med Educ. 2016;50(1):132–149. 26695473 10.1111/medu.12927

[26] Jennings JD, Quinn C, Ly JA, et al. Orthopaedic surgery resident financial literacy: an assessment of knowledge in debt, investment, and retirement savings. Am Surg. 2019;85(4):353–358. 31043194

[27] Rohlfing J, Navarro R, Maniya OZ, et al. Medical student debt and major life choices other than specialty. Med Educ Online. 2014;19(1):25603. 25391976 10.3402/meo.v19.25603 PMC4229497

